# Review of the planthopper genus *Neohemisphaerius* (Hemiptera, Fulgoroidea, Issidae) with description of one new species from China

**DOI:** 10.3897/zookeys.568.6700

**Published:** 2016-02-23

**Authors:** Zheng-Guang Zhang, Zhi-Min Chang, Xiang-Sheng Chen

**Affiliations:** 1 School of Life Sciences, Jinggangshan University, Ji’an, Jiangxi, 343009, P.R. China; 2 Institute of Entomology, Guizhou University, Guiyang, Guizhou Province 550025, P.R. China

**Keywords:** Fulgoromorpha

## Abstract

The planthopper genus *Neohemisphaerius* Chen, Zhang & Chang, 2014 (Hemiptera: Fulgoroidea: Issidae) is reviewed to include 3 species: *Neohemisphaerius wugangensis* Chen, Zhang & Chang, 2014 (China: Hunan), *Neohemisphaerius yangi* Chen, Zhang & Chang, 2014 (China: Guangdong) and *Neohemisphaerius guangxiensis* **sp. n.** (China: Guangxi). A revised generic diagnosis is given. The new species is described and all species illustrated. A key to these three species is also given. The species *Neohemisphaerius signifer* (Walker) is transferred back to *Hemisphaerius* as *Hemisphaerius signifer* Walker, **comb. revived**.

## Introduction

The genus *Neohemisphaerius* was erected by Chen, Zhang & Chang, 2014 for two new species (*Neohemisphaerius wugangensis* and *Neohemisphaerius yangi*) and *Neohemisphaerius signifer* Walker, 1851, from China. In this paper, one new species of the genus *Neohemisphaerius* is described and illustrated from China, the generic characteristics are redefined and a checklist and key to the known species of the genus are provided. In addition, *Neohemisphaerius signifer* is removed from the genus; its placement was based on the identification by Fennah (1956) which has proven erroneous when compared to studied images of the type in the Natural History Museum, London. This type, which differs from *Neohemisphaerius* in lacking a median carina on the frons and in having the anteclypeus flat and hindwings well developed, is returned to *Hemisphaerius* as comb. revived, pending further studies. The non-type specimen from China figured by Fennah (1956) as *Neohemisphaerius signifer* belongs to an unknown species.

## Material and methods

The morphological terminology of the head and body follows Chan and Yang (1994), and the terminology of male genitalia follows Gnezdilov (2003). The genital segments of the examined specimens were macerated in 10% KOH and drawn from preparations in glycerin jelly using a light microscope. Photographs of the specimens were made using Zeiss stereo Discovery V8. Microscope with Zeiss Axio Cam HRc camera, images were produced using the software Axion Vision V4.8.2.0 and edited and enhanced using Adobe Photoshop CS4.0.

The type specimens of the new species are deposited in School of Life Sciences, Jinggangshan University.

## Taxonomy

### Genus Neohemisphaerius

Taxon classificationAnimaliaHemipteraIssidae

Chen, Zhang & Chang, 2014

Neohemisphaerius Chen, Zhang & Chang, 2014: 80

#### Type species.

*Neohemisphaerius wugangensis* Chen, Zhang & Chang, 2014.

#### Diagnosis.

Body hemispherical, head including eyes wider than pronotum. Vertex about 2.5–3.1 times wider than long, anterior margin more or less straight, posterior margin angulately concave, disc depressed, edges carinated. Frons longer than broad, with median carina, lateral margins slightly elevated. Clypeus convex on disc, distinctly tapering to apex. Pronotum depressed on disc, with two central pits, edges carinated. Mesonotum subtriangular, without median and lateral carinae. Forewings hemispherical, claval suture present. Hind wings rudimentary, veins indistinct. Hind tibiae with 2 lateral teeth. Spinal formula of the hind leg (9,10)-(4,5)-2.

#### Distribution.

China (Guangdong, Guangxi, Hunan)

#### Discussion.

The genus *Neohemisphaerius* is similar to *Hemisphaerius* Schaum, 1850 and *Gergithus* Stål, 1870, but it differs from *Hemisphaerius* in: frons with median carina; clypeus with a hump-shaped process in middle and forewings with claval suture present. It differs from *Gergithus* in: frons with median carina; forewings with claval suture present; hind wings rudimentary, shorter than half length of forewings.

#### Key to species of genus *Neohemisphaerius*

**Table:** 

1	Forewings (Figs 1–2, 4–5) pale brown, with two black patches at costal margin; aedeagus (Figs 9–10) dorsally with a hump-shaped process, each side with birdhead-shaped processes	***Neohemisphaerius guangxiensis* sp. n.**
–	Forewings (Figs 13–14) yellowish, with extensive black markings; aedeagus not as above	**2**
2	Frons (Fig. 16) with distinct median carina; anal tube (Chen et al. 2014: fig. 2–35: H) in dorsal view with apical margin sinuate; aedeagus (Chen et al. 2014: figs 2–35: M, K) ventrally with short hooks, shorter than 1/5 length of aedeagus; spinal formula of hind leg10-4-2	***Neohemisphaerius wugangensis***
–	Frons (Fig. 20) with obscure median carina; anal tube (Chen et al. 2014: fig. 2-36: H) in dorsal view apical margin round; aedeagus (Chen et al. 2014: figs 2–36: L, K) ventrally with long hooks, longer than half length of aedeagus; spinal formula of hind leg 9-5-2	***Neohemisphaerius yangi***

**Figures 1–6.**
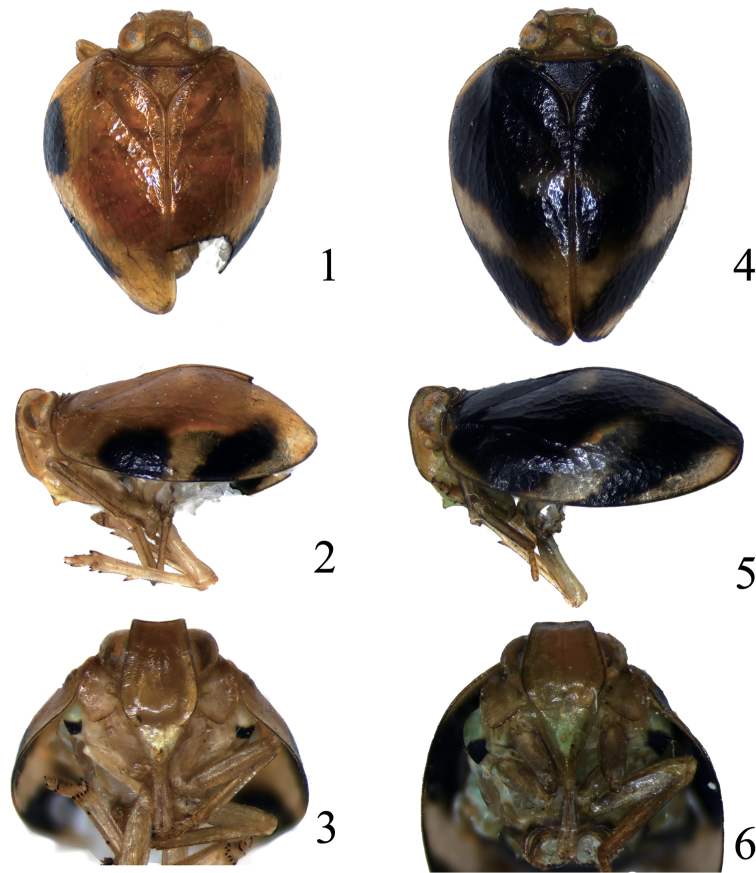
*Neohemisphaerius guangxiensis* sp. n. **1** Adult (male), dorsal view **2** Adult (male), in lateral view **3** Frons and clypeus (male), in front view **4** Adult (female), in dorsal view **5** Adult (female), in lateral view **6** Frons and clypeus (female), in front view.

**Figures 7–12.**
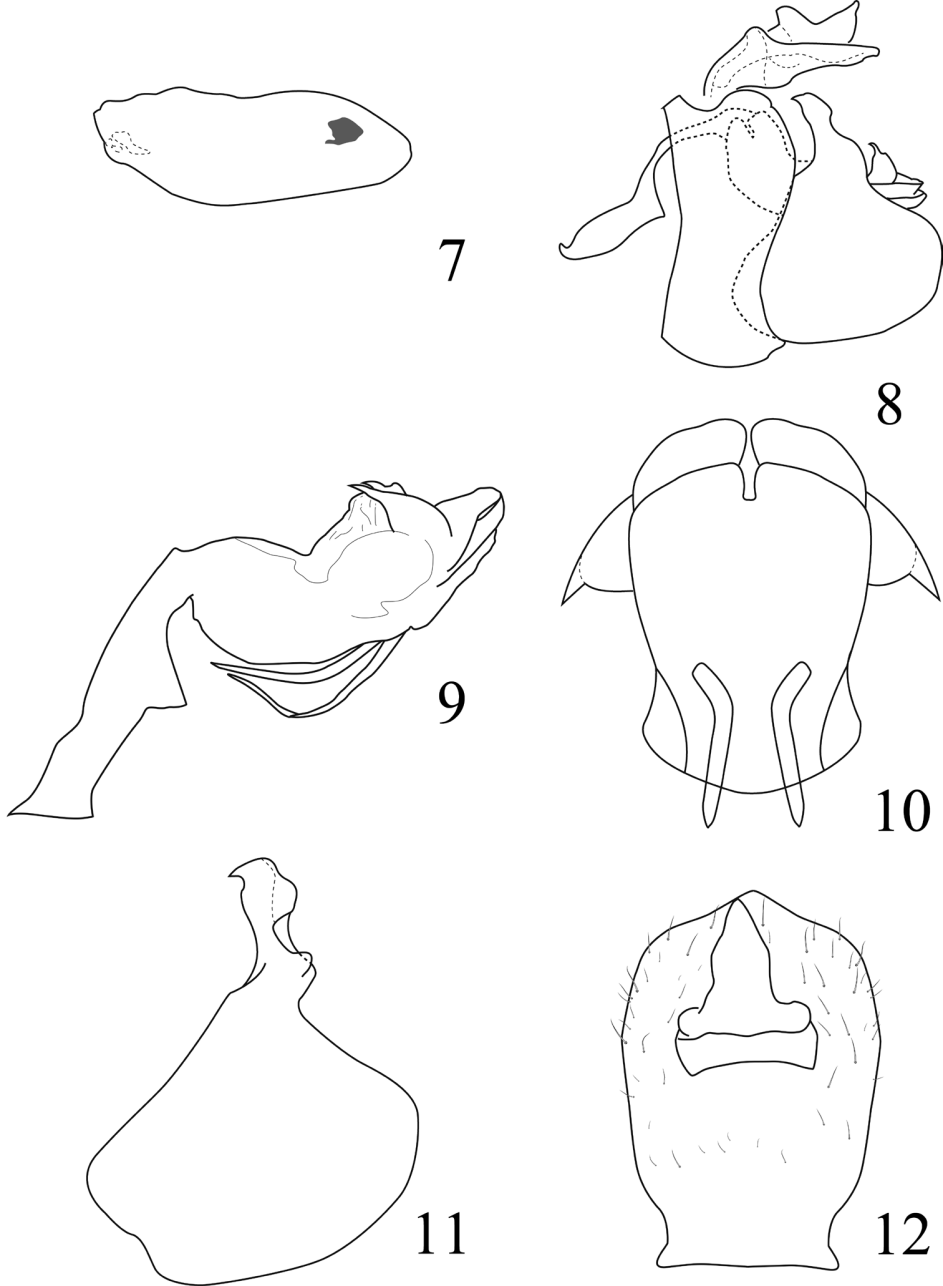
*Neohemisphaerius guangxiensis* sp. n. **7** Hind wing **8** Male genitalia, in lateral view **9** Aedeagus, in left view **10** Aedeagus, ventral view **11** Genital style, in profile view **12** Anal tube, in dorsal view.

**Figures 13–16.**
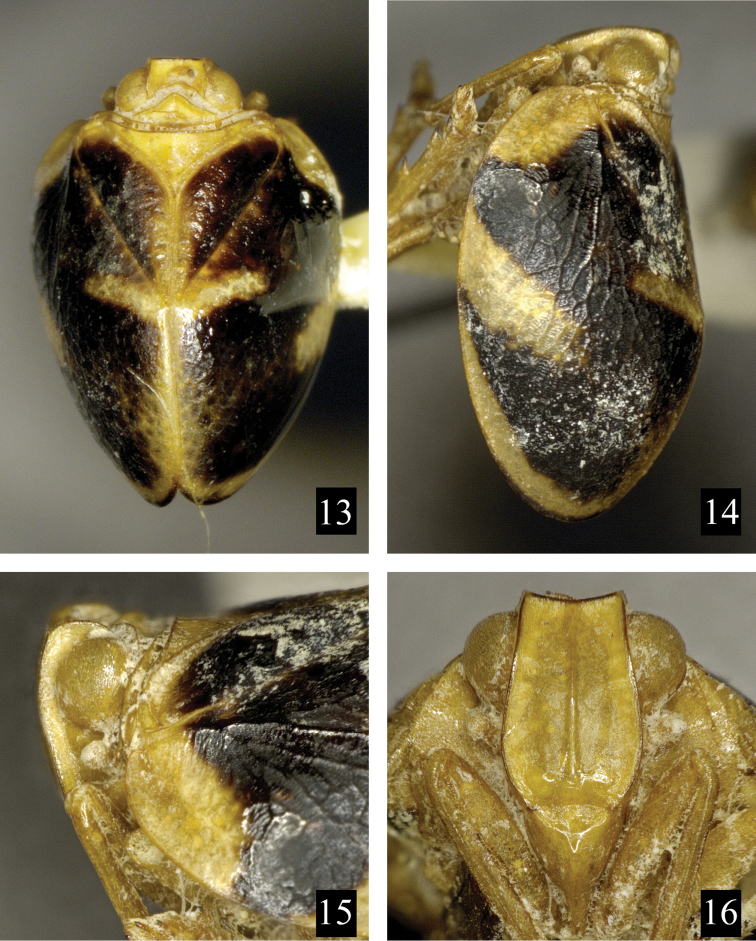
*Neohemisphaerius wugangensis* Chen, Zhang & Chang, 2014. **13** Adult (male), in dorsal view **14** Adult (male), in lateral view **15** Head (male), in lateral view **16** Frons and clypeus (male), in front view.

**Figures 17–20.**
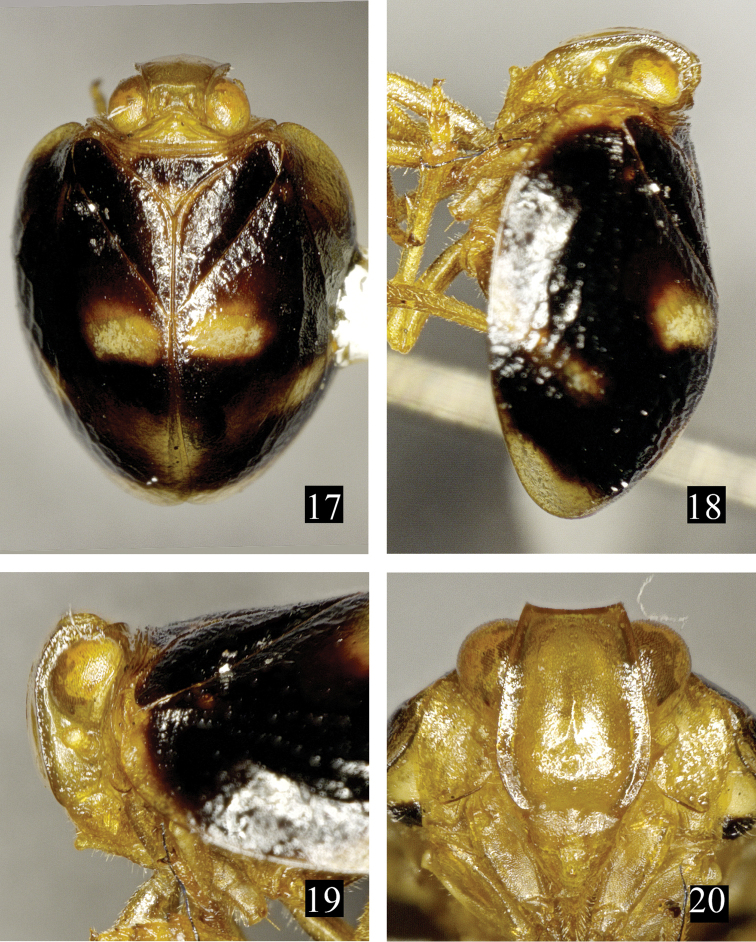
*Neohemisphaerius yangi* Chen, Zhang & Chang, 2014. **17** Adult (male), in dorsal view **18** Adult (male), in lateral view **19** Head (male), in lateral view **20** Frons and clypeus (male), in front view.

### Neohemisphaerius guangxiensis sp. n.

Taxon classificationAnimaliaHemipteraIssidae

http://zoobank.org/9362854B-F612-4BDB-8A71-4859C8E76C4F

Figs 1–6 , 7–12

#### Type material.

Holotype: ♂, China: Guangxi, Maoershan National Nature Reserve (E110°27'56.9", N25°54'43.5"), 1470 m, 18 July 2015, Z.G. Zhang; paratypes: 2 ♂♂, 5 ♀♀, same data as holotype.

#### Description.

Body length (from apex of vertex to tip of forewing): male 4.63 mm, female 5.21 mm; Forewing: male 4.12 mm, female 4.60 mm.

#### Coloration.

Male: Vertex (Fig. 1) and frons (Fig. 3) brown, edges dark brown. Clypeus (Fig. 3) pale yellowish, rostrum (Fig. 3) dark brown, antenna brown (Figs 2–3). Pronotum (Fig. 1) brown with pale brown on disc, mesonotum (Fig. 1) brown with lateral angles dark brown. Forewings (Figs 1–2) brown with black markings near costal margin, hind wing pale brown. Legs pale brown. Female: Clypeus (Fig. 6) pale green near base, rostrum (Fig. 6) dark brown. Pronotum (Fig. 4) dark brown, mesonotum black brown. Forewings (Figs 4–5) with extensive irregular black markings, costal margin with pale brown spots at base and apex, pale stripe arising from middle of costal margin oblique to suture.

#### Head and thorax.

Vertex (Fig. 1) quadrangular, about 3.14 times wider than long, anterior margin straight, posterior margin angulately concave. Frons (Fig. 3) narrow at base, widest between eyes, about 1.36 times longer than broad, median carina present, distinctly convex above frontoclypeal suture. Clypeus (Fig. 3) with a hump-like process. Pronotum (Fig. 1) with posterior margin straight, depressed on disc, with two central pits. Mesonotum (Fig. 1) subtriangular, about 1.94 times longer in midline than the length of pronotum. Forewings (Figs 1–2) hemispherical, claval suture present, with longitudinal veins. Hind wings rudimentary, veins obscure. Hind tibiae with 2 lateral teeth. Spinal formula of the hind leg (9,10)-(4,5)-2.

#### Male genitalia.

Anal tube (Fig. 12) relatively short, oval in dorsal view. Anal column relatively long, located at 1/3 basad of anal tube. Pygofer (Fig. 8) in lateral view, with anterior margin moderately concave, posterior margin raised near base. Aedeagus dorsally (Fig. 9) with a hump-shaped process near mid-length, each side with a bird-head-shaped process at 1/3 distance from apex, process acute apically, directed cephalad; dorso-lateral lobes obtuse apically, aedeagus ventrally with a pair of convergent hook-like processes, apical margin of ventral lobe (Fig. 10) with a notch in middle. Style (Fig. 11) with a strongly convex hind margin and capitulum narrowing apically.

#### Etymology.

The specific name refers to the locality, Guangxi province, China.

#### Host plant.

Unknown.

#### Distribution.

China (Guangxi).

**Figure 21.**
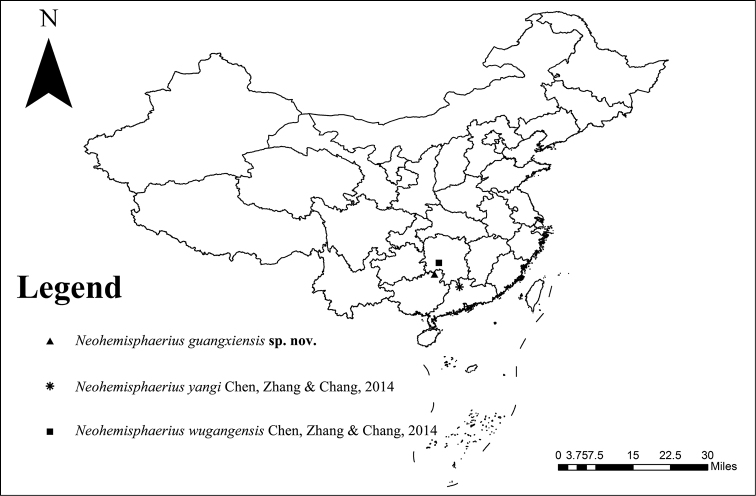
Geographic distribution of *Neohemisphaerius* species in China.

#### Remarks.

This species is similar to *Neohemisphaerius wugangensis*, but differs in: (i) Anal tube (Fig. 12) longer than broad, with apical margin not expanded (in *wugangensis* anal tube about as long as broad, apical margin expanded (see Chen et al. 2014: figs 2–35: H); (ii) Aedeagus (Fig. 9) with a bird-head-shaped subapical process in each side, ventrally with pairs of long hooks near mid-length (in *wugangensis* processes of aedeagus different and ventrally with pairs of short hooks 1/3 from base); (iii) Apical margin of ventral lobe (Fig. 10) with a notch in middle (in *wugangensis* ventral lobe with apical margin convex in middle (see Chen et al. 2014: fig. 2–35: K).

### Neohemisphaerius wugangensis

Taxon classificationAnimaliaHemipteraIssidae

Chen, Zhang & Chang, 2014

Figs 13–16

Neohemisphaerius wugangensis Chen, Zhang & Chang, 2014: 80: figs 2–35.

#### Material examined.

1♂4♀♀, Yunshan National Forest Park, Wugang city, Hunan Province, China

### Neohemisphaerius yangi

Taxon classificationAnimaliaHemipteraIssidae

Chen, Zhang & Chang, 2014

Figs 17–20

Neohemisphaerius yangi Chen, Zhang & Chang, 2014: 83: figs 2–36.

#### Material examined.

2♂♂7♀♀, Nanling National Nature Reserve, Guangdong Province, China.

## Supplementary Material

XML Treatment for Neohemisphaerius

XML Treatment for Neohemisphaerius guangxiensis

XML Treatment for Neohemisphaerius wugangensis

XML Treatment for Neohemisphaerius yangi
